# Corneal nerve and endothelial cell damage in patients with transient ischemic attack and minor ischemic stroke

**DOI:** 10.1371/journal.pone.0213319

**Published:** 2019-03-15

**Authors:** Hoda Gad, Adnan Khan, Naveed Akhtar, Saadat Kamran, Ahmed El-Sotouhy, Soha R. Dargham, Ioannis N. Petropoulos, Georgios Ponirakis, Ashfaq Shuaib, Leopold J. Streletz, Rayaz A. Malik

**Affiliations:** 1 Department of Medicine, Weill Cornell Medicine-Qatar, Doha, Qatar; 2 Institute of Neurosciences, Hamad Medical Corporation, Doha, Qatar; 3 Department of Radiology, Hamad Medical Corporation, Doha, Qatar; 4 Biostatistics, Epidemiology & Biomathematics Research Core, Weill Cornell Medicine-Qatar, Doha, Qatar; 5 Department of Medicine, University of Alberta, Edmonton, Canada; 6 Institute of Cardiovascular Medicine, University of Manchester, Manchester, United Kingdom; Ehime University Graduate School of Medicine, JAPAN

## Abstract

**Objective:**

To determine if corneal confocal microscopy can identify corneal nerve and endothelial cell abnormalities and may be useful in the prognostication of patients with transient ischemic attack [1] or minor ischemic stroke (IS).

**Methods:**

Thirty-six patients admitted with TIA (n = 14) or minor IS (n = 22) underwent transcranial Doppler evaluation and corneal confocal microscopy and were compared with 18 healthy controls.

**Results:**

Corneal nerve fiber density (*P* = 0.002), branch density (*P* = 0.004) and fiber length (*P* = 0.004) were significantly lower in patients with TIA or minor IS compared to controls, with no difference between patients with TIA and minor IS. Endothelial cell density (*P* = 0.003) was lower and endothelial cell area (*P* = 0.003) and perimeter (*P* = 0.006) were significantly higher in patients with TIA or minor IS compared to controls, with no difference between patients with TIA and minor IS. There were no differences in corneal nerve or endothelial cell morphology between patients with and without abnormal cerebrovascular reactivity. HbA1c was independently associated with CNFL, and endothelial cell polymegathism and pleomorphism were associated with both HbA1c and total cholesterol.

**Conclusion:**

Corneal confocal microscopy identifies corneal nerve fiber loss and endothelial cell abnormalities in patients with TIA and minor IS and independent associations with HbA1c and cholesterol.

## Introduction

Stroke is associated with high fatality rates and major disability in survivors [2]. Transient Ischemic Attack [1] and minor ischemic stroke (IS) share similar pathophysiology to stroke [3]. Although, the ABCD2 score has been used to prognosticate the risk of subsequent stroke [4], a meta-analysis showed that it does not reliably discriminate patients at low or high risk of recurrent stroke [5]. Similarly, neuroimaging may enhance the prognostic ability following TIA and minor stroke. However, recent analyses of patients with TIA or minor IS show that white matter lesions are associated with disability at 90 days, but not with stroke progression or stroke recurrence [6], and micro bleeds predict neither 90-day outcome or recurrence [7].

Cerebral auto-regulation assures hemodynamic integrity of the cerebral circulation [8] and maintains cerebral blood flow (CBF) [9]. In addition to arterial blood pressure, intracranial pressure and cerebral venous pressure may affect auto regulation and CBF [10]. Whilst impaired cerebral auto regulation is associated with poor functional and prognostic outcomes in patients with ischemic stroke [9], only a third of patients with acute ischemic stroke have impaired cerebral auto regulation and it does not relate to stroke type or severity [11].

Corneal confocal microscopy (CCM) is a noninvasive ophthalmic imaging technique, which allows rapid, high-resolution imaging of the cornea. We have pioneered this technique to identify axonal loss in patient with diabetes [12], impaired glucose tolerance [13, 14] and other peripheral neuropathies [15]. CCM can also detect corneal nerve loss in Parkinson’s disease [16], amyotrophic lateral sclerosis [17] and multiple sclerosis [18]. Recently, in patients with major ischemic stroke we have shown a significant reduction in corneal nerves [19] and abnormalities in endothelial cells [20].

### Hypothesis

We hypothesize that patients with TIA and minor IS will have evidence of corneal nerve and endothelial cell abnormalities which will aid in prognostication of patients with TIA and minor stroke.

## Methods

Forty patients with TIA or minor IS, aged between 18-80-year-old and able to provide consent were enrolled in the study. The diagnosis of TIA or minor ischemic stroke was confirmed clinically and radiologically by neurologists and neuroradiologists using AHA criteria [21]. Patients with craniocerebral trauma, hypertensive encephalopathy, brain tumor, atrial fibrillation or taking anticoagulants were excluded. Three patients were excluded as they were found to be TIA mimics and one had cerebral venous sinus thrombosis. Thirty-six patients underwent Transcranial Doppler Ultrasound (TCD) and Corneal Confocal Microscopy (CCM). Ethical approvals were obtained from the Institutional Review Boards of Hamad General Hospital and Weill Cornell Medicine in Qatar.

### Corneal confocal microscopy

**Fig 1:**
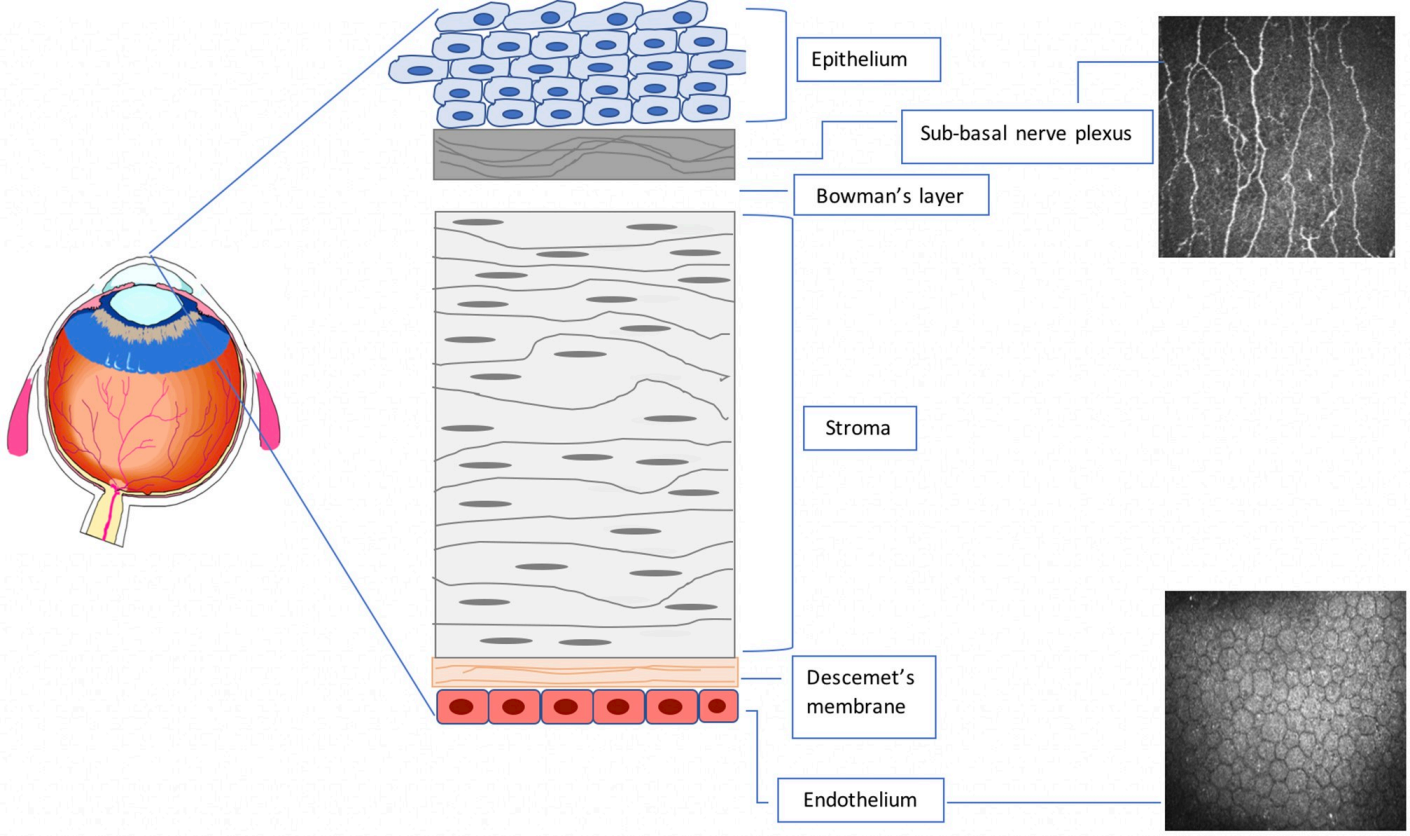
Schematic of the corneal layers indicating the level at which corneal nerve and endothelial cell images are captured.

All patients underwent CCM (Heidelberg Retinal Tomograph III Rostock Cornea Module; Heidelberg Engineering GmbH, Heidelberg, Germany). To perform the CCM examination, local anesthetic (0.4% benoxinate hydrochloride; Chauvin Pharmaceuticals, Chefaro, United Kingdom) was used to anesthetize both eyes, and Viscotears (Carbomer 980, 0.2%, Novartis, United Kingdom) was used as the coupling agent between the cornea and the CCM [22]. The examiners captured central sub-basal nerve plexus images using the section mode (Fig 1). On the basis of depth, contrast, focus, and position, 6 images per patient were selected [23]. Corneal nerve fiber density (CNFD), corneal nerve branch density (CNBD), corneal nerve fiber length (CNFL) and corneal nerve fiber tortuosity (CNFT) were analysed manually using CCMetrics (M. A. Dabbah, ISBE, University of Manchester, Manchester, United Kingdom) [12] and the investigator was blinded to the diagnosis. Corneal endothelial cell density, area, perimeter and degree of polymegathism (cell size variability) and pleomorphism (cell shape variability) were quantified using automated CEAS software [24].

### Transcranial doppler ultrasound

Blood flow in the right and left middle cerebral arteries [25] was measured using a trans-temporal approach. Basal and peak flow velocities and cerebrovascular reactivity to hypercapnia was measured by the Breath-Holding Index (BHI) [26].

$$\frac{Mean\;MCAV-Mean\;MCAV\;baseline}{Mean\;MCAV\;at\;baseline}\times \frac{100}{Seconds\;of\;breath\;holding}\geq 0.69$$

### Statistical analysis

Statistical analyses were performed using IBM SPSS Statistics software Version 25. Normality of the data was assessed using the Shapiro-Wilk test and by visual inspection of the histogram and a normal Q-Q plot. Data are expressed as mean and SD for the normally distributed variables and as median and range for the skewed variables. Inferential analyses were conducted for the corneal nerve and endothelial cell outcomes using both parametric (T-test and ANOVA) and non-parametric (Mann-Whitney U and Kruskal–Wallis) tests, with Bonferroni adjustment. To investigate the association between risk factors for corneal nerve and endothelial cell parameters, Pearson and Spearman correlation were performed as appropriate. Multiple linear regression analysis was conducted to evaluate the independent association between corneal nerve and endothelial cell parameters and their covariates. Significance level was set at α = 0.05. Prism 6 (version 6.0g; Graphpad software Inc, CA) was used to plot the charts.

## Results

### Clinical and metabolic characteristics

The clinical and metabolic characteristics are summarized in Table 1. Thirty-six patients with TIA (n = 14) and minor IS (n = 22) were compared with 18 age-matched healthy controls without diabetes, hypertension or previous TIA/stroke. Of the 36 patients with TIA and IS, based on HbA1c and history, 13 had no diabetes; 9 had pre-diabetes and 14 had Type 2 diabetes. There was no significant difference in age (*P* = 0.241), HbA1c (*P* = 0.243), total cholesterol (*P* = 0.092); LDL-C (*P* = 0.309); HDL-C (*P* = 0.105); TG (*P* = 0.192) or body mass index (*P* = 0.195) between control subjects and patients with TIA or minor IS. Systolic blood pressure (SBP) was significantly higher (*P* = 0.012) in patients with TIA or minor IS compared to controls.

**Table 1:** Clinical, metabolic and CCM parameters in patients with TIA, minor IS and healthy controls.

	Control	TIA	Minor IS	*P-Value*
**Clinical characteristics**				
Age (years)	43.39 ± 13.73	47.36 ± 8.71	48.84 ± 8.77	0.241
NIHSS	N/A	1 ± 1	2 ± 2	0.001*
Mean BHI	N/A	0.52 ± 0.57	0.47 ± 0.55	0.787
HbA_1c_ (%)	5.6 ± 0.30	6.0 ± 1.10	7.0 ± 2.70	0.243
Total cholesterol (mmol/L)	3.95 ± 1.93	4.32 ± 1.02	5.13 ± 1.40	0.092
LDL-C (mmol/L)	2.96 ± 1.07	2.51 ± 0.93	3.12 ± 1.23	0.309
HDL-C (mmol/L)	1.12 ± 0.21	0.91 ± 0.21	0.97 ± 0.23	0.105
TG (mmol/L)	1.2 ± 0.70	2.1 ± 1.50	2.3 ± 1.80	0.192
SBP (mmHg)	120.9 ± 12.40	147.57 ± 24.83^†^	143.45 ± 23.02^†^	0.012*
DBP (mmHg)	75.1 ± 7.29	85.0 ± 14.81	88.23 ± 13.90	0.040
BMI (kg/m^2^)	25.97 ± 1.84	26.46 ± 2.35	28.34 ± 4.61	0.195
**CCM**				
CNFD (fibers/mm^2^)	38.18 ± 7.85	30.12 ± 8.32^†^	28.86 ± 8.05^†^	0.002*
CNBD (branches/mm^2^)	69.79; 87.50	44.79; 119.79	37.5; 108.33^†^	0.004^‡^
CNFL (mm/mm^2^)	21.55 ± 4.19	16.80 ± 5.07^†^	16.41 ± 5.20^†^	0.004*
CNFT (TC)	0.04; 0.10	0.03; 0.07	0.03; 0.09	0.186
ECD (cells/mm^2^)	3633 ± 176.00	3411 ± 408.00	3366 ± 229.00^†^	0.003*
ECA (μm^2^)	222 ± 11.00	240 ± 30.00^†^	241 ± 18.00^†^	0.003*
ECP (μm)	53.0 ± 1.00	55.0 ± 4.00^†^	55.0 ± 2.00^†^	0.006*
EC polymegathism (%)	52.0 ± 5.00	51.0 ± 3.00	52.0 ± 5.00	0.825
EC pleomorphism (%)	34.0 ± 5.00	34.0 ± 4.0	35.0 ± 6.0	0.894

All results were expressed as mean ± SD, except CNBD and CNFT expressed as median; range. TIA: Transient ischemic attack; IS: Ischemic Stroke; LDL-C: Low-density lipoprotein-cholesterol; HDL-C: High-density lipoprotein-cholesterol; TG: Triglycerides; SBP: Systolic blood pressure; DBP: Diastolic blood pressure; BMI: Body mass index; CNFD: Corneal nerve fiber density; CNBD: Corneal nerve branch density; CNFL: Corneal nerve fiber length; CNFT: Corneal nerve fiber tortuosity; ECD: Endothelial cell density; ECA: Endothelial cell area; ECP: Endothelial cell perimeter; EC: Endothelial cell. NIHSS and mean BHI were not assessed for the control group.

* Statistically significant differences between groups using ANOVA.

‡ Statistically significant difference between groups using Kruskal-Wallis test.

†Post hoc results differ significantly from the control group after adjustment for multiple comparisons using Bonferroni correction (*P*<0.02).

### Corneal confocal microscopy

**Fig 2:**
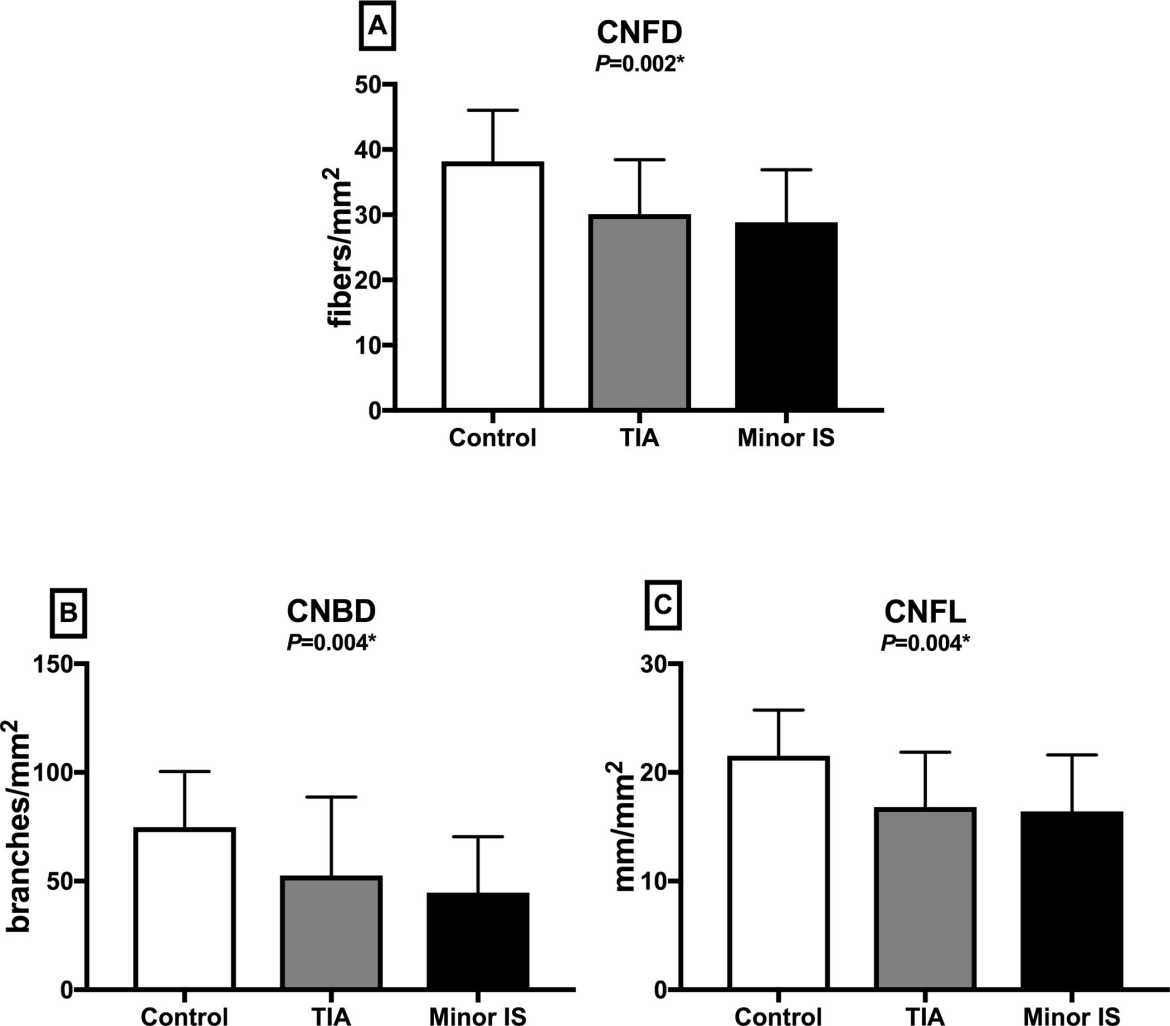
Corneal nerve fiber parameters in control subjects and patients with TIA and minor IS. (A) CNFD: Corneal nerve fiber density; (B) CNBD: Corneal nerve branch density, (C) CNFL: Corneal nerve fiber length; Data are expressed as mean ± SD. TIA: Transient Ischemic Attack; IS: Ischemic stroke.

**Fig 3:**
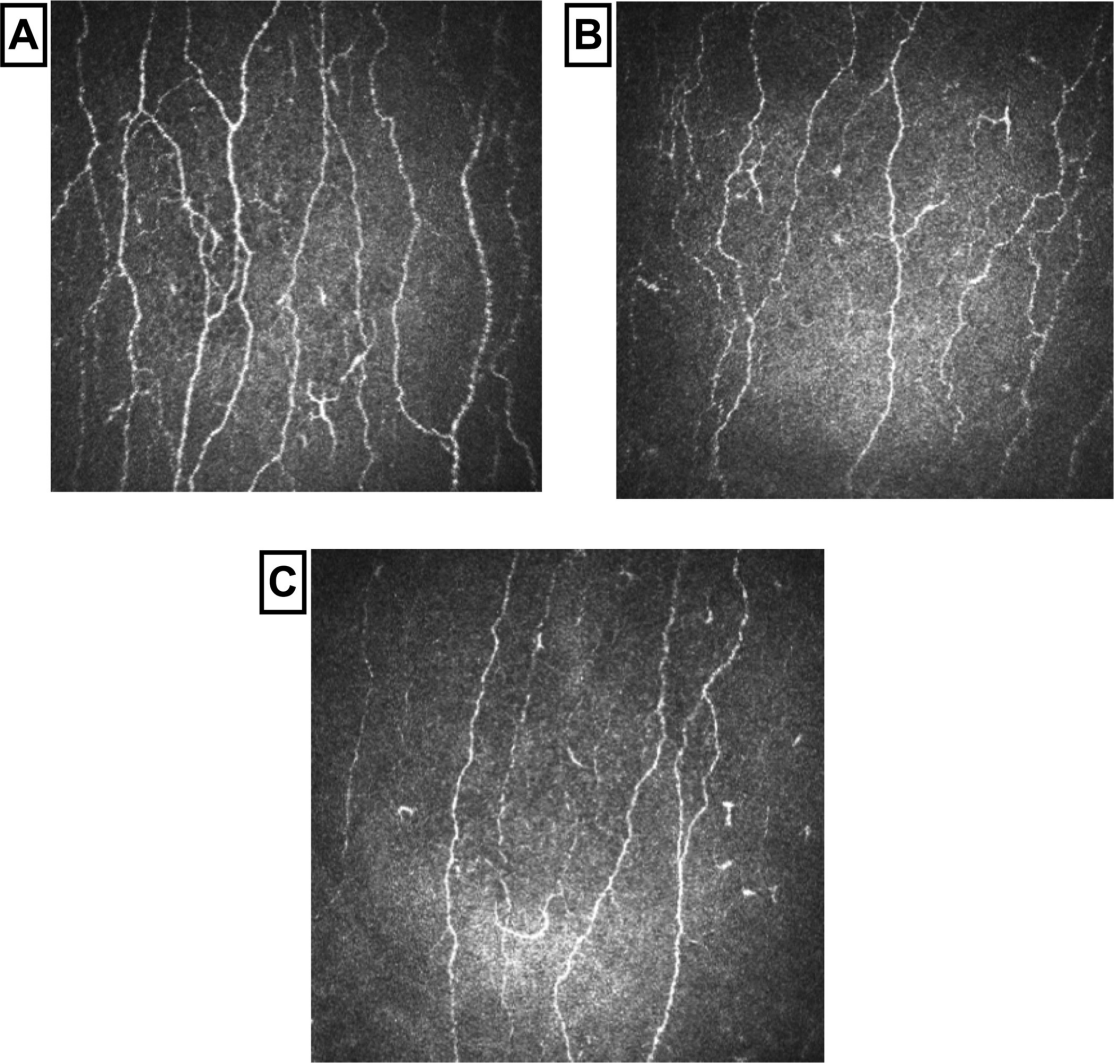
Images of corneal sub-basal nerve plexus. Control subject (A), patient with TIA (B) and a patient with minor IS (C).

**Fig 4:**
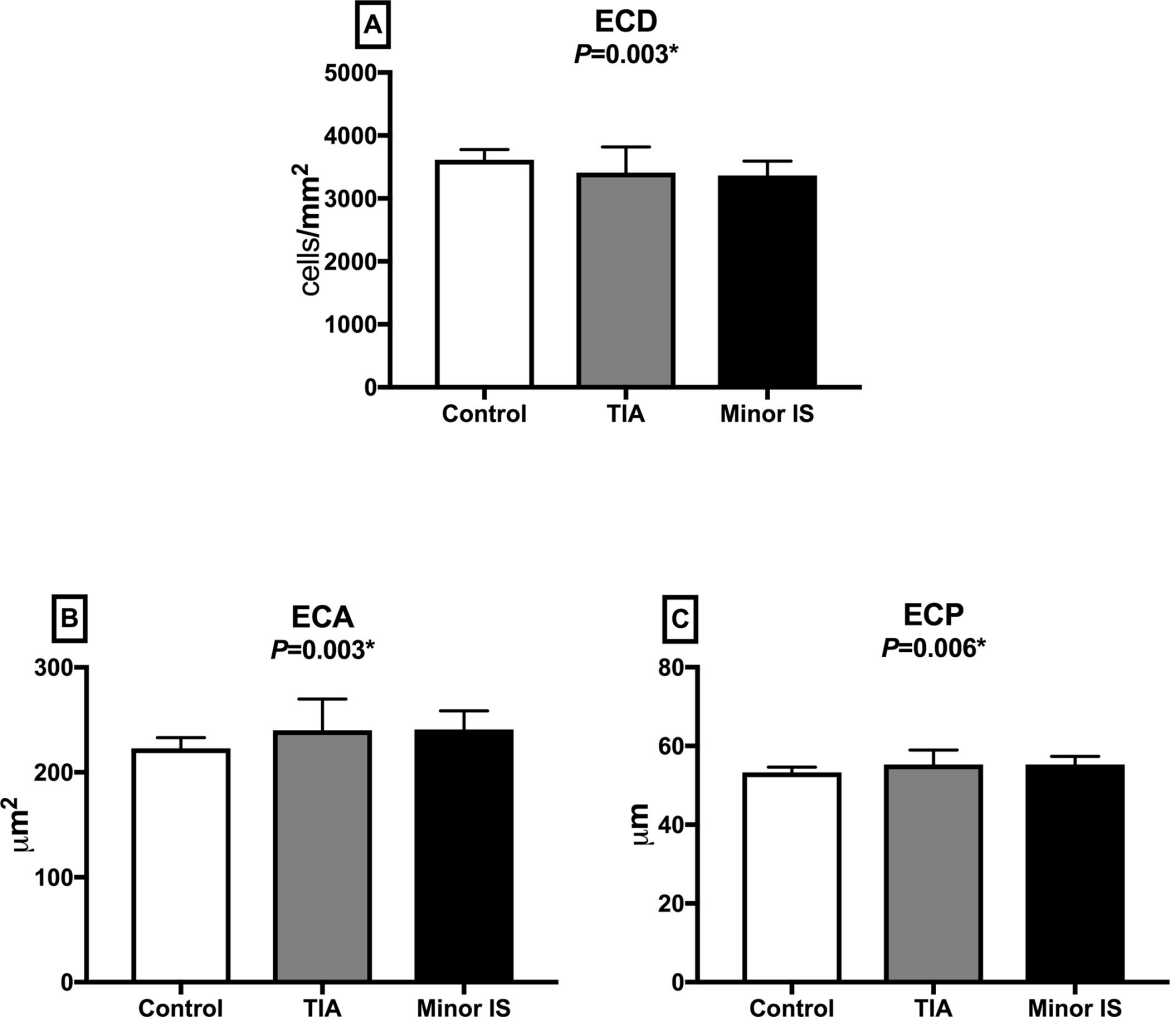
Corneal endothelial cell parameters in control subjects and patients with TIA and minor IS. (A) ECD: Endothelial cell density; (B) ECA: Endothelial cell area; (C) ECP: Endothelial cell perimeter. Data are expressed as mean ± SD. TIA: Transient Ischemic Attack; IS: Ischemic stroke.

**Fig 5:**
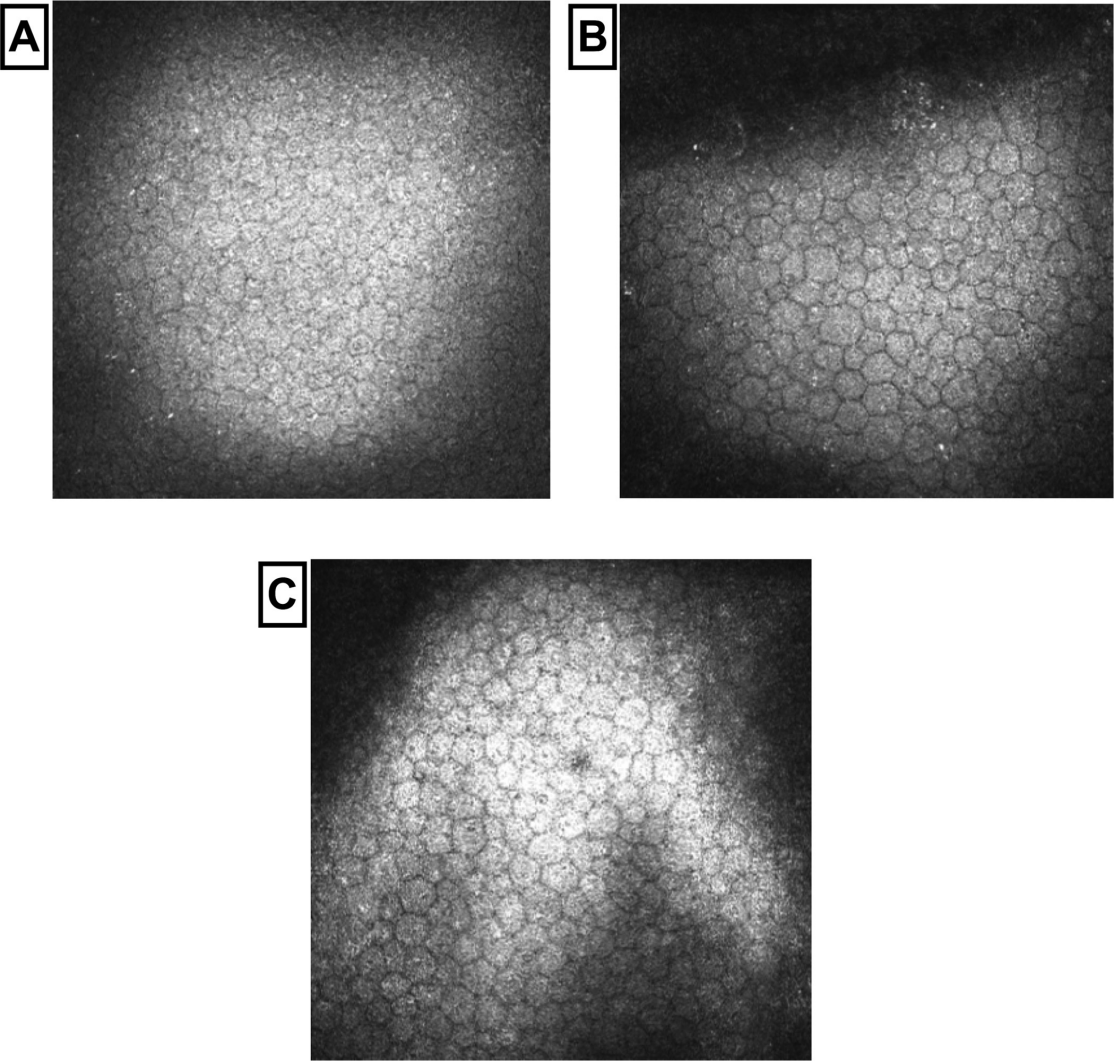
Images of corneal endothelium. Control subject (A), patient with TIA (B) and a patient with minor IS (C).

CNFD (*P* = 0.002), CNBD (*P* = 0.004) and CNFL (*P* = 0.004) were significantly lower in patients with TIA or minor IS compared to controls, with no difference between patients with TIA or minor IS (Table 1, Figs 2 and 3). Endothelial cell density (*P* = 0.003) was lower and endothelial cell area (*P* = 0.003) and perimeter (*P* = 0.006) were significantly higher with no difference in the degree of polymegathism (*P* = 0.825) and pleomorphism (*P* = 0.894) between patients with TIA or minor IS compared to controls and no difference between patients with TIA or minor IS (Table 1, Figs 4 and 5). There was no difference in CNFD (*P* = 0.77), CNBD (*P* = 0.08), CNFL (*P* = 0.45), endothelial cell density (*P* = 0.44), endothelial cell area (*P* = 0.41), perimeter (*P* = 0.42), polymegathism (*P* = 0.95), and pleomorphism (*P* = 0.90), between participants without diabetes, pre-diabetes and diabetes.

### Cerebrovascular reactivity

64% of patients with TIA (n = 14) and 68% with minor IS (n = 22) had abnormal BHI. Comparing patients with normal and abnormal BHI there was no significant difference in: CNFD (30.52 ± 7.10; 28.80 ± 8.49; *P* = 0.58); CNBD (33.85 and 64.58; 41.93 and 127.08; *P* = 0.36); CNFL (16.71 ± 4.11; 16.49 ± 5.51; *P* = 0.91); CNFT (0.03 and 0.09; 0.03 and 0.07; *P* = 0.93); ECD (3399.84 ± 344.58; 3371.92 ± 274.36; *P* = 0.80); ECA (239.58 ± 24.89; 241.02 ± 21.19; *P* = 0.86); ECP (55.23 ± 2.78; 55.3 ± 2.56; *P* = 0.94); percentage with polymegathism (52.49 ± 4.68; 51.32 ± 4.14; *P* = 0.47) and pleomorphism (35.03 ± 7.01; 34.14 ± 4.90; *P* = 0.67).

#### Correlation

NIHSS at presentation correlated with CNFD (*r* = 0.364, *P* = 0.031) and CNFL (*r* = 0.345, *P* = 0.046). There was no correlation between corneal nerve and endothelial cell parameters and BHI or age.

#### Multiple linear regression

There were independent associations between some corneal nerve and endothelial cell parameters with age, HbA_1c_ and total cholesterol (Table 2). There was a significant association between HbA_1c_ with CNFL (*B* = -0.768, *P* = 0.04) and endothelial cell pleomorphism (*B* = -1.261, *P* = 0.001). Total cholesterol was associated with endothelial cell polymegathism (*B* = -1.628, *P* = 0.016) and pleomorphism (*B* = 2.637, *P* = 0.001). Age was significantly associated with endothelial cell pleomorphism (*B* = 0.412, *P* = <0.001). BHI was not associated with CNFL (*B* = -0.137, *P* = 0.217); CNFD (*B* = 2.1, *P* = 0.456); endothelial cell density (*B* = -124.545, *P* = 0.306); endothelial cell area (*B* = 9.607, *P* = 0.295); endothelial cell perimeter (*B* = 1.309, *P* = 0.228), polymegathism (*B* = 1.758, *P* = 0.272) or pleomorphism (*B* = -1.829, *P* = 0.321). CNBD and CNFT were skewed, therefore they were not included in the multiple regression analysis.

**Table 2:** Independent risk factors for altered corneal nerve and endothelial cell parameters in patients with TIA and minor IS.

	B	95% CI	SE	*P-Value*
**CNFD (fibers/mm**^**2**^**)**				
Age (years)	-0.199	(-0.551–0.153)	0.1795	0.268
Mean BHI	2.1	(-3.42–7.619)	2.8162	0.456
HbA_1c_ (%)	-1.038	(-2.222–0.147)	0.6044	0.086
Total cholesterol (mmol/L)	1.514	(-0.908–3.936)	1.2359	0.22
TG (mmol/L)	-1.315	(-2.914–0.284)	0.8157	0.107
**CNFL (mm/mm**^**2**^**)**				
Age (years)	-0.137	(-0.36–0.08)	0.1113	0.217
Mean BHI	0.61	(-2.81–4.03)	1.746	0.727
**HbA**_**1c**_ **(%)**	**-0.768**	**(-1.50 - -0.03)**	**0.3747**	0.04^*^
Total cholesterol (mmol/L)	0.681	(-0.82–2.18)	0.7662	0.374
TG (mmol/L)	-0.558	(-1.55–0.43)	0.5057	0.270
**ECD (cells/mm**^**2**^**)**				
Age (years)	-9.881	(-25.172–5.41)	7.8018	0.205
Mean BHI	-124.545	(-363.039–113.949)	121.6829	0.306
HbA_1c_ (%)	14.899	(-33.596–63.395)	24.7431	0.547
Total cholesterol (mmol/L)	-13.324	(-113.824–87.175)	51.2761	0.795
TG (mmol/L)	14.665	(-51.387–80.717)	33.7005	0.663
**ECA (μm**^**2**^**)**				
Age (years)	0.704	(-0.45–1.858)	0.5888	0.232
Mean BHI	9.607	(-8.392–27.606)	9.1834	0.295
HbA_1c_ (%)	-0.879	(-4.539–2.781)	1.8673	0.638
Total cholesterol (mmol/L)	1.223	(-6.361–8.808)	3.8698	0.752
TG (mmol/L)	-0.758	(-5.743–4.227)	2.5434	0.766
**ECP (μm)**				
Age (years)	0.051	(-0.086–0.187)	0.0696	0.468
Mean BHI	1.309	(-0.818–3.436)	1.085	0.228
HbA_1c_ (%)	0.002	(-0.431–0.434)	0.2206	0.994
Total cholesterol (mmol/L)	0.01	(-0.886–0.906)	0.4572	0.982
TG (mmol/L)	-0.108	(-0.697–0.481)	0.3005	0.72
**EC Polymegathism (%)**				
Age (years)	-0.198	(-0.399–0.003)	0.1027	0.054
Mean BHI	1.758	(-1.381–4.896)	1.6012	0.272
**HbA**_**1c**_ **(%)**	**0.637**	**(-0.001–1.275)**	**0.3256**	**0.050**
**Total cholesterol (mmol/L)**	**-1.628**	**(-2.951 - -0.306)**	**0.6747**	0.016^*^
TG (mmol/L)	0.628	(-0.241–1.497)	0.4435	0.157
**EC Pleomorphism (%)**				
**Age (years)**	**0.412**	**(0.18–0.643)**	**0.118**	0.001^*^
Mean BHI	-1.829	(-5.437–1.78)	1.841	0.321
**HbA**_**1c**_ **(%)**	**-1.261**	**(-1.995 - -0.527)**	**0.3743**	0.001^*^
**Total cholesterol (mmol/L)**	**2.637**	**(1.116–4.157)**	**0.7758**	0.001^*^
TG (mmol/L)	-0.321	(-1.32–0.679)	0.5099	0.529

BHI: Breath holding index; HbA1c: Glycated hemoglobin; TG: Triglycerides; CNFL: Corneal nerve fiber length; CNFD: Corneal nerve fiber density; ECD: Endothelial cell density; ECA: Endothelial cell area; ECP: Endothelial cell perimeter; EC: Endothelial cells.

## Discussion and conclusions

This is the first study to demonstrate corneal nerve and endothelial cell pathology in patients with TIA or minor IS, extending our previous findings in patients with major stroke [19, 20]. Diabetes, hypertension, smoking, dyslipidemia [27–29], obesity [25] and metabolic syndrome [30] are known risk factors for stroke and are linked to cerebral white matter lesions and silent lacunar brain infarcts [31], but have limited prognostic value for recurrent stroke in patients with TIA and minor IS [4]. Impaired cerebral reactivity has been associated with the risk of subsequent stroke in patients with TIA [32, 33], and smoking, hypertension, diabetes and cholesterol are related to altered CBF in patients with TIA and minor stroke [31, 34]. Endothelial dysfunction is involved in the pathophysiology of TIA [35] and lacunar stroke [36] and has been implicated in the development of silent lacunar infarcts and white matter lesions [37]. It may also act as an independent predictor for a recurrent ischemic event [38, 39].

The corneal endothelium has traditionally been thought to play a role in primarily regulating the passage of nutrients and metabolic waste to and from the cornea [40], however, it also shows thrombogenic potential after exposure to extracellular matrix and collagen [41]. We have previously demonstrated a reduction in corneal endothelial cell density in patients with diabetes [42, 43]. We have also recently developed an automated image analysis system to quantify corneal endothelial cell morphology and shown reduced corneal endothelial cell density and hypertrophy in patients with diabetes [24]. Given that we found corneal nerve and endothelial cell abnormalities in patients with TIA and minor stroke, we assessed for associations with cerebrovascular reactivity and risk factors for stroke. We show no difference in corneal endothelial cell and nerve morphology between patients with and without abnormal cerebrovascular reactivity, suggesting alternate mechanisms driving these two abnormalities in patients with cerebrovascular disease.

Contrary to our previous studies in subjects with impaired glucose tolerance and diabetes (12, 13), we failed to demonstrate a difference in corneal nerve and endothelial cell pathology between participants without diabetes, pre-diabetes and diabetes. This was despite an association between endothelial cell polymegathism and pleomorphism with total cholesterol and HbA1c and between CNFL and HbA1c. Indeed, we have previously shown a loss of corneal nerves in subjects with impaired glucose tolerance (IGT) and type 2 diabetes with a major stroke compared to controls, but no difference between participants with IGT and T2DM, despite an association between corneal nerve morphology with HbA_1c_ and triglycerides [19]. We can only attribute this lack of difference to an as yet unidentified confounding bias in this population with cerebrovascular disease, the relatively small cohort size and the influence of concurrent medication. CNFL and CNFD correlated directly with the severity of stroke at presentation, arguing that alterations in corneal nerve morphology are not related to the acute event. Age correlated with CNFL, which agrees with a number of previous studies [44, 45].

A limitation of this study is the small sample size of younger, predominantly South Asian patients, which might limit the generalizability of our study findings. However, this is the first study to show an abnormality in corneal nerves and endothelial cells in patients with TIA and minor stroke, and extend our recent findings in patients with major stroke. There is a need for larger, longitudinal studies to assess the prognostic value of corneal nerve and endothelial cell imaging in relation to recurrent TIA or stroke.

## Supporting information

S1 Dataset (XLSX)
