# Correction: Corneal nerve and endothelial cell damage in patients with transient ischemic attack and minor ischemic stroke

**DOI:** 10.1371/journal.pone.0217672

**Published:** 2019-05-28

**Authors:** Hoda Gad, Adnan Khan, Naveed Akhtar, Saadat Kamran, Ahmed El-Sotouhy, Soha R. Dargham, Ioannis N. Petropoulos, Georgios Ponirakis, Ashfaq Shuaib, Leopold J. Streletz, Rayaz A. Malik

The following information is missing from the Funding statement: This article was funded by the Qatar National Library. The funder had no role in study design, data collection and analysis, decision to publish, or preparation of the manuscript.
